# Comparing COVID-19 literacy and vaccine hesitancy among health care workers, including oral health professionals, in Japan

**DOI:** 10.1038/s41405-024-00282-9

**Published:** 2025-01-04

**Authors:** Fujimi Ueno, Satoru Haresaku, Hidechika Iino, Tomoaki Taguchi, Ryuji Sakagami, Koichiro Matsumoto, Kotaro Kudo, Masahiro Yoneda, Akiko Chishaki, Kenji Okada

**Affiliations:** 1 Fukuoka Nursing College, Graduate School of Nursing, 2-15-1 Tamura, Sawara-ku, Fukuoka, 814-0193 Japan; 2 Department of Nursing, Fukuoka Nursing College, Graduate School of Nursing, 2-15-1 Tamura, Sawara-ku, Fukuoka, 814-0193 Japan; 3 Fukuoka College of Health Sciences, 2-15-1 Tamura, Sawara-ku, Fukuoka, 814-0193 Japan; 4 Fukuoka Dental College Medical and Dental Hospital, 2-15-1 Tamura, Sawara-ku, Fukuoka, 814-0193 Japan; 5 International University of Health and Welfare, School of Health Sciences at Fukuoka Department of Nursing, 137-1 Enokizu, Okawa, Fukuoka 831-8501 Japan; 6 Section of General Dentistry, Department of General Dentistry, Fukuoka Dental College, 2-15-1 Tamura, Sawara-ku, Fukuoka, 814-0193 Japan

**Keywords:** Occupational health, Dental public health

## Abstract

**Background:**

Oral health professionals should have good COVID-19 vaccine literacy as should physicians and nurses. However, little is known about COVID-19 literacy and vaccine hesitancy among oral health professionals in Japan.

**Aims:**

This study aimed to investigate the status of COVID-19 literacy and vaccine hesitancy among oral health professionals by comparing them with other healthcare workers (HCWs).

**Methods:**

To compare these differences, a self-administered questionnaire-based survey of 596 staff members was conducted in March 2023 at schools, an affiliated hospital, and elderly care facilities after the staff who wished to receive it completed the fifth dose of COVID-19 vaccinations. Comparison between the recognition levels and number of vaccine doses among the HCWs were examined using the Kruskal–Wallis tests. Defining the third or lower doses of COVID-19 vaccine as vaccine hesitancy, factors associated with the hesitancy were examined using logistic regression analysis.

**Results:**

In total, 408 (68.5%) participants were enrolled for the study. Dental hygienists’ COVID-19 literacy levels were lower compared to those of nurses and physicians. Dentists received a significantly lower number of vaccine doses than did physicians. Vaccine hesitancy was positively associated with younger age and higher concern about the adverse effects of the vaccine, but it was not associated with occupation.

**Conclusion:**

Interventions to improve the low COVID-19 literacy and high COVID-19 vaccine hesitancy among oral health professionals, particularly young ones, are needed for COVID-19 and future pandemics.

## Introduction

Owing to the intensive scientific investments in vaccine development, COVID-19 vaccines have rapidly become available, first in developed countries, followed by the rest of the world [1]. In Japan, the first COVID-19 vaccine dose was administrated in March 2021, and the seventh dose was administered in November 2023 [2]. As of 14 November 2023, 80.9% of the Japanese population had received at least one dose of the vaccine, and 67.4% had received at least three doses [2].

Recent studies have confirmed the efficacy of COVID-19 vaccines, including the booster dose, in preventing people from contracting the disease and in reducing the risk of hospitalizations and deaths [3–8]; therefore, the vaccinations have been among the most important public health methods for preventing COVID-19 infection [9].

Vaccine hesitancy poses a challenge in promoting the vaccination against COVID-19. The World Health Organization (WHO) defines vaccine-hesitant people as individuals who possess varying levels of indecisiveness regarding particular vaccines or overall immunisation [10]. Further, WHO identified vaccine hesitancy as one of the top 10 threats to public health in 2019 [11]. Many studies reported COVID-19 vaccine hesitancy among general populations [12], health care workers (HCWs) [13], and oral health professionals [14]. COVID-19 literacy, meaning understanding COVID-19 and making informed decisions based on that understanding [15, 16], is a key element required for successful control of the COVID-19 pandemic. The association between this literacy and COVID-19 vaccination hesitancy has also been reported in many countries [12]. However, to the best of our knowledge, only one Greek study has compared the literacy and hesitancy regarding COVID-19 vaccines between dentists and other health professionals [17].

Oral health professionals are at the frontline of infection, particularly with respiratory viruses such as the novel coronavirus [18]. The Japanese government granted an extraordinary permission for dentists to deliver vaccines, aiming to support largescale vaccination efforts without further compromising access to medical care, and from May to the end of July 2021, a cumulative total dose of 721,471 COVID-19 vaccines were provided to community residents by a total of 12,727 dentists [19]. Similarly, some parts of the United States expanded the scope of dental practice to enable dentists to provide COVID-19 vaccines to community residents [20]. Therefore, it is suggested that oral health professionals should have good COVID-19 vaccine literacy as well as physicians and nurses.

Therefore, this study aimed to investigate the status of COVID-19 literacy and vaccine hesitancy among oral health professionals by comparing them with other HCWs.

## Materials and methods

### Survey sample and data collection

This cross-sectional questionnaire survey was conducted at the facilities of a school corporation. The school corporation is located in the suburbs of Fukuoka City and has a dental, dental hygienist, nursing schools, as well as an affiliated hospital and three affiliated elderly facilities. The study participants included the staff in facilities located on the same ground, except for an elderly facility located approximately 1 km away from the other facilities. A self-administered questionnaire survey was conducted from 15 February to 8 March 2023 after the fifth dose of the COVID-19 vaccine was administered for all staff who requested it at the health centre adjacent to the hospital, from 28 September to 9 December 2022. They managed to schedule dates and times in advance for the vaccinations and received the first through fifth doses of the vaccines at the health centre, free of charge, during working hours.

The staff included physicians, dentists, nurses, dental hygienists (DH), care workers (CW), other HCWs, and non-HCWs. Other HCWs, such as pharmacists, radiologic technicians, therapists, dental technicians, laboratory technicians, optometric technicians, and clinical psychologists, were small groups (less than 10 persons) and were excluded from this study’s analysis to limit the number of groups for comparison analysis. Non-HCWs included academic and administrative staff without professional qualifications for medical care.

The questionnaire was distributed to staff across various departments in the school corporation. On the front page of the questionnaire, the aim of the project and voluntary nature of the study were explained. The second page was the consent form. Those who agreed to participate checked ‘I agree to participate in the research’ on the consent form, completed the questionnaire, and submitted it in the box provided by the researcher in each department. Participants were informed that their participation was voluntary, and those who did not want to participate could put the questionnaire in the box without checking the consent disclaimer. After the deadline for submitting the questionnaires in the box had passed, the researchers collected them.

This study was conducted in accordance with the guidelines of the Declaration of Helsinki and approved by the Ethics Committee of Fukuoka Gakuen, Fukuoka, Japan (No. 615).

### Methods of measurement

This study’s questionnaire had sociodemographic factors included sex, age group (20–29, 30–39, 40–49, 50–59, more than 60 years), occupation, workplace, underlying medical conditions, and COVID-19 vaccination experience and number of doses (Supplementary Table), and had 13 questions based on validated scales and indicators, to measure confidence and literacy regarding COVID-19 and its vaccine. [21–23]. Items regarding COVID-19 literacy and confidence included the recognition of correct and incorrect information regarding COVID-19 (three and two items, respectively), the recognition of having a lot of knowledge (one item), the recognition of experiencing a new type of COVID-19 infection (one item), and being worried about contracting COVID-19 (one item). The remaining five items concerned COVID-19 vaccine literacy and confidence. They included confidence in the preventive effects of the COVID-19 vaccine (three items), concern about adverse reactions to the COVID-19 vaccine (one item), and recognition of the possibility of experiencing fever or swelling at the vaccination site after COVID-19 vaccinations (one item). To measure the level of confidence and literacy regarding COVID-19 and the vaccine, the following scores in points were assigned: strongly disagree = 1, disagree = 2, neither agree nor disagre = 3, agree = 4, and strongly agree = 5.

To measure COVID-19 Vaccine hesitancy, the number of vaccine doses that each participant received was assessed. The answer choices ranged from no dose to the fifth dose. Having a lower number of doses was defined as having a higher hesitancy to the vaccine. In addition, the number of doses was divided into 2 groups, ‘third or lower doses’ and ‘fourth and fifth doses’, because the distributions of those with less than three doses were too small, and that for those with five doses was too large. Therefore, ‘third or lower doses’ was defined as hesitant and ‘fourth or fifth doses’ was defined as acceptant.

To identify the factors associated with hesitancy, hesitant (third or lower doses) and acceptant (fourth and fifth doses) were chosen as dependent variables, and hesitant and acceptant were replaced with scores of 1 and 0, respectively. Sex, age group, having underlying medical conditions, occupation, and recognition of the COVID-19 Vaccine were chosen as independent variables. ‘Male’ in sex, ‘No’ in having underlying medical conditions, ‘20-29 years’ in age group, ‘Physician’ in occupation, as well as ‘Strongly agree’ and ‘Agree’ in the recognition regarding COVID-19 Vaccine were chosen as references and replaced to score 0.

### Statistical analysis

A chi-squared test was performed to test the differences in the characteristics and distribution of the number of doses in the occupation group. A Kruskal–Wallis test was performed to compare the occupation group’s literacy and confidence levels of COVID-19 and the vaccine and their number of COVID-19 vaccine doses. After the analysis, Bonferroni’s multiple-comparison test was performed to compare the differences in the literacy and confidence levels and number of doses between the occupation groups, controlling for Type I errors arising from the multiple comparisons. A logistic regression analysis was used to determine the associated factors with COVID-19 vaccine hesitancy. The tests were performed at a significance level of <5%. IBM SPSS Statistics (version 28.0; IBM Corporation, Armonk, NY, USA) was used for the statistical analysis.

## Results

In total, 456 participants responded to the questionnaire, with a response rate of 76.5%. This study analysed data from 408 (239 males, 165 females, and 4 unknown sex) participants, excluding 44 persons in the small occupation groups and 4 persons in the unknown occupation.

Table 1 presents the distribution of these characteristics. Almost all nurses and dental hygienists (DHs) were female; however, the majorities in the other groups were male. The majority (approximately 70%) of dentists and DHs were under 40 years of age, while the majority of participants in the other groups were over 40 years of age. The majority of health professionals, physicians, dentists, nurses, and DHs worked at the hospital, while almost all CWs worked at the elderly care facility. Approximately 30% of the physicians and dentists worked at the dental school, 31.0% of the nurses worked at the nursing school, and 14.6% of the DHs worked at the dental hygiene school. More than 40% of the CWs had underlying medical conditions, while less than 20% of the dentists and DHs had medical conditions. There were significant differences in age, sex, workplace, and underlying medical conditions between the occupational groups.

**Table 1:** Distribution of characteristics according to occupation.

	Total *n* = 408	Physician *n* = 29	Dentist *n* = 127	Nurse *n* = 84	DH^a^ *n* = 48	CW^b^ *n* = 18	non-HCW^c^ *n* = 102	*p*-Value^*^
	*n* (%)	*n* (%)	*n* (%)	*n* (%)	*n* (%)	*n* (%)	*n* (%)	
Sex								
Male	165 (40.8)	18 (62.1)	82 (65.6)	4 (4.9)	0 (0.0)	8 (44.4)	53 (52.0)	<0.001
Female	239 (59.2)	11 (37.9)	43 (34.4)	78 (95.1)	48 (100.0)	10 (55.6)	49 (48.0)	
Age (years)								
20–29	78 (19.4)	0 (0.0)	31 (24.6)	11 (13.8)	24 (50.0)	2 (11.1)	10 (9.8)	<0.001
30–39	119 (29.6)	10 (35.7)	55 (43.7)	14 (17.5)	11 (22.9)	1 (5.6)	28 (27.5)	
40–49	100 (24.9)	7 (25.0)	16 (12.7)	27 (33.8)	9 (18.8)	9 (50.0)	32 (31.4)	
50–59	65 (16.2)	5 (17.9)	14 (11.1)	20 (25.0)	3 (6.3)	4 (22.2)	19 (18.6)	
60+	40 (10.0)	6 (21.4)	10 (7.9)	8 (10.0)	1 (2.1)	2 (11.1)	13 (12.7)	
Workplace								
Hospital	206 (50.6)	15 (53.6)	85 (67.0)	48 (57.1)	40 (83.4)	0 (0.0)	18 (17.6)	<0.001
Dental school	102 (25.1)	9 (32.1)	37 (29.1)	0 (0.0)	0 (0.0)	0 (0.0)	56 (54.9)	
Dental hygiene school	20 (4.9)	1 (3.6)	4 (3.1)	0 (0.0)	7 (14.6)	1 (5.6)	7 (6.9)	
Nursing school	45 (11.1)	2 (7.1)	1 (0.8)	26 (31.0)	0 (0.0)	0 (0.0)	16 (15.7)	
Elderly care facility	34 (8.4)	1 (3.6)	0 (0.0)	10 (11.9)	1 (2.1)	17 (94.4)	5 (4.9)	
Having underlying medical conditions								
No	322 (79.5)	22 (75.9)	104 (83.2)	57 (68.7)	44 (91.7)	10 (55.6)	85 (83.3)	0.002
Yes	83 (20.5)	7 (24.1)	21 (16.8)	26 (31.3)	4 (8.3)	8 (44.4)	17 (16.7)	

*Chi-squared test

^a^Dental Hygienist.

^b^Care Worker.

^c^Non-Health worker (Administration or academic staff without any health care qualification).

Table 2 presents a comparison of the COVID-19 literacy and confidence levels. The levels of recognising correct information regarding COVID-19 (Q1 and Q2) were high (more than 4.0 points) while those of recognising incorrect information (Q7 and Q8) were low. The levels of concern about getting COVID-19 and the recognition level of contracting a new type of COVID-19 were 3.8 and 3.3 points, respectively. After the Bonferroni correction test, the recognition level of the DHs in Q6 was significantly lower than that of the physicians, and in Q8, their recognition level was significantly lower than that of the nurses.

**Table 2:** Comparison of the literacy and confidence level^a^ regarding COVID-19 according to occupation.

	Total *n* = 408	1. Physician *n* = 29	2. Dentist *n* = 127	3. Nurse *n* = 84	4. DH^b^ *n* = 48	5. CW^c^ *n* = 18	6. non-HCW^d^ *n* = 102	*p*-Value^*^	Occupation number pair^e^	*p*-Value^**^
	Mean (SD)	Mean (SD)	Mean (SD)	Mean (SD)	Mean (SD)	Mean (SD)	Mean (SD)			
Q1. COVID-19 easily spreads from person to person.	4.2 (0.8)	4.4 (0.7)	4.2 (0.8)	4.4 (0.7)	4.1 (0.7)	4.2 (0.8)	4.2 (0.8)	0.117		
Q2. COVID-19 is more severe in people over 65 years old and those with chronic illnesses.	4.0 (0.8)	4.0 (0.7)	4.1 (0.7)	3.9 (0.8)	4.0 (0.7)	4.1 (0.7)	4.0 (0.8)	0.676		
Q3. I am worried about getting COVID-19.	3.8 (1.0)	3.6 (1.1)	3.9 (0.9)	3.8 (1.0)	3.9 (0.9)	3.8 (1.0)	3.7 (1.0)	0.521		
Q4. I may get a new type of COVID-19.	3.3 (0.9)	3.4 (1.0)	3.3 (0.9)	3.2 (0.9)	3.2 (0.7)	3.4 (0.8)	3.1 (0.9)	0.416		
Q5. I know a lot about COVID-19	3.2 (0.8)	3.4 (0.7)	3.2 (0.9)	3.3 (0.8)	2.9 (0.8)	3.3 (0.9)	3.0 (0.8)	0.043		
Q6. Many people with COVID-19 have mild illnesses.	2.8 (1.1)	3.5 (1.1)	3.0 (1.1)	2.7 (1.0)	2.5 (0.9)	2.5 (0.9)	2.6 (1.1)	0.001	1-4	0.019
Q7. Anyone with COVID-19 will have symptoms.	2.3 (1.0)	1.9 (0.8)	2.3 (1.0)	2.3 (1.1)	2.5 (0.8)	2.1 (0.8)	2.2 (0.9)	0.042		
Q8. Once you have COVID-19, you cannot get it again.	1.7 (0.8)	1.4 (0.6)	1.8 (0.8)	1.5 (0.7)	2.0 (0.9)	1.7 (0.9)	1.7 (0.7)	0.007	1-4 3-4	0.032 0.012

*Kruskal–Wallis test; **Bonferroni-adjusted *p*-value.

^a^Strongly disagree = 1, disagree = 2, neither agree nor disagree = 3, agree = 4, and strongly agree = 5.

^b^Dental Hygienist.

^c^Care Worker.

^d^Non-Health Care Workers (Administration or academic staff without any health care qualification).

^e^Occupation number pairs with statistically significant difference after Bonferroni correction.

Table 3 presents a comparison of the recognition levels regarding COVID-19 vaccine according to occupations. Overall, the recognition level of the possibility of fever or swelling at the vaccination site was high (4.3 points). On comparing the recognition levels among occupation groups, significant differences were found in all question items except for Q12. The DHs had the highest level of concern about the adverse reactions of the vaccination (4.2 point) among the occupation groups, and it was significantly higher than that of the physicians (*p* = 0.016). Conversely, the DHs’ confidence level regarding the preventive effects of the COVID-19 vaccine was the lowest (3.4 point) among the occupation groups, and it was significantly lower than that of the non-HCWs (*p* = 0.012).

**Table 3:** Comparison of the recognition level^a^ regarding COVID-19 vaccine according to occupations.

	Total *n* = 408	1. Physician *n* = 29	2. Dentist *n* = 127	3. Nurse *n* = 84	4. DH^b^ *n* = 48	5. CW^c^ *n* = 18	6. non-HCW^d^ *n* = 102	*p*-Value^*^	Occupation number pair^e^	*p*-Value^**^
	Mean (SD)	Mean (SD)	Mean (SD)	Mean (SD)	Mean (SD)	Mean (SD)	Mean (SD)			
Q9. You may experience fever or swelling at the vaccination site after receiving the COVID-19 vaccine.	4.3 (0.7)	4.1 (0.7)	4.2 (0.8)	4.5 (0.7)	4.5 (0.6)	4.3 (0.8)	4.4 (0.6)	0.044		
Q10. I am concerned about adverse reactions to the COVID-19 vaccine.	3.9 (0.8)	3.5 (0.8)	3.8 (0.9)	3.9 (0.9)	4.2 (0.6)	3.9 (0.8)	3.9 (0.8)	0.024	1-4	0.006
Q11. Prevention of COVID-19 in vaccinated persons.	3.7 (0.9)	3.8 (0.9)	3.8 (0.9)	3.7 (0.9)	3.4 (1.0)	3.6 (0.5)	3.9 (0.8)	0.014	4-6	0.012
Q12. Prevent family members and friends of the inoculated person from contracting COVID-19.	3.7 (0.9)	3.7 (1.0)	3.9 (0.9)	3.7 (0.9)	3.5 (0.9)	3.4 (0.8)	3.7 (0.9)	0.139		
Q13. Prevent the spread of COVID-19 in the vaccinated person’s area.	3.7 (0.9)	3.8 (0.9)	3.8 (0.9)	3.8 (0.8)	3.4 (1.0)	3.3 (0.9)	3.8 (0.8)	0.031		

*Kruskal–Wallis test; **Bonferroni-adjusted *p*-value.

^a^Strongly disagree = 1, disagree = 2, neither agree nor disagree = 3, agree = 4, and strongly agree = 5.

^b^Dental Hygienist.

^c^Care Worker.

^d^Non-Health Care Workers (Administration or academic staff without any health care qualification).

^e^Occupation number pairs with statistically significant difference after Bonferroni correction.

Table 4 presents the distribution and number of COVID-19 vaccine doses according to occupations. The percentages according to the number of doses were 2.7% for no dose and 0.2%, 2.0, 19.7%, 19.7%, 27.5%, and 47.9% for the first, second, third, fourth, and fifth doses, respectively. Regarding the comparison of distribution according to occupation, more than 30% of dentists and DHs received the third or lower doses while the percentages in the other groups were less than 25%. The distribution had no significant differences among the occupation groups (*p* = 0.200). The median (Interquartile range: IQR) number of doses was highest for physicians and nurses [5.0 (4.0-5.0)], and lowest for dentists [4.0 (3.0-5.0)]. The number of doses differed significantly among the occupation groups (*p* = 0.005), and the number of doses for dentists was significantly lower than that for physicians (*p* = 0.005).

**Table 4:** Distribution and number of COVID-19 vaccine doses according to occupation.

	Total *n* = 408	1. Physician *n* = 29	2. Dentist *n* = 127	3. Nurse *n* = 84	4. DH^b^ *n* = 48	5. CW^c^ *n* = 18	6. non-HCW^d^ *n* = 102			
	*n* (%)	*n* (%)	*n* (%)	*n* (%)	*n* (%)	*n* (%)	*n* (%)	*p*-Value^*^		
Number COVID-19 vaccine doses										
No dose	11 (2.7)	0 (0.0)	7 (5.5)	1 (1.2)	1 (2.1)	0 (0.0)	2 (2.0)	0.200		
First dose	1 (0.2)	0 (0.0)	0 (0.0)	1 (1.2)	0 (0.0)	0 (0.0)	0 (0.0)			
Second dose	8 (2.0)	0 (0.0)	4 (3.1)	1 (1.2)	1 (2.1)	0 (0.0)	2 (2.0)			
Third dose	80 (19.7)	4 (13.8)	33 (26.0)	6 (7.1)	14 (29.2)	3 (16.7)	20 (19.8)			
Fourth dose	112 (27.5)	8 (27.6)	33 (26.0)	26 (31.0)	10 (20.8)	7 (38.9)	28 (27.7)			
Fifth dose	195 (47.9)	17 (58.6)	50 (39.4)	49 (58.3)	22 (45.8)	8 (44.4)	49 (48.5)			
	**Mean (SD)**	**Mean (SD)**	**Mean (SD)**	**Mean (SD)**	**Mean (SD)**	**Mean (SD)**	**Mean (SD)**	*p*-Value^**^	**Occupation number pair**^**e**^	*p*-Value^***^
Median (IQR) dose of COVID-19 vaccine										
	4.0 (4.0–5.0)	5.0 (4.0–5.0)	4.0 (3.0–5.0)	5.0 (4.0–5.0)	4.0 (3.0–5.0)	4.0 (4.0–5.0)	4.0 (4.0–5.0)	0.008	1–2	0.005

*Chi-squared test; **Kruskal–Wallis test; ***An occupation pair with statistically significant difference after Bonferroni correction.

^a^Dental Hygienist.

^b^Care Worker.

^c^Non-Health worker (Administration or academic staff without any health care qualification).

Table 5 presents the factors associated with COVID-19 vaccine hesitancy, based on logistic regression analysis. Regarding participants’ characteristics, younger age was significantly associated with hesitancy (third or lower vaccine doses). The odds ratio (95% Confidence Interval: CI) of the ‘60+’ group to ‘20–29’ group was 0.24 (0.06–0.93). The low recognition (‘strong disagree’, ‘disagree’, and ‘neither’) of experiencing fever or swelling at the vaccination site (Q9) and the high recognition (‘strong agree’ and ‘agree’) of concern about adverse reactions (Q10) were significantly associated with hesitancy. The odds ratios (95% CI) of the high recognition group to the low recognition group were 0.41 (0.17–0.99) in Q9 and 2.66 (1.23–5.74) in Q10.

**Table 5:** Associated factors with Covid-19 vaccine hesitancy^a^ by logistic regression analysis.

		95% confidence interval		
	Odds ratio	Upper	Lower	*p*-value^*^
Sex				
Male =0	1.00	–	–	
Female =1	0.77	0.42	1.41	0.402
Age (years)				
20–29 (Reference)	1.00	–	–	
30–39	0.80	0.40	1.57	0.510
40–49	0.56	0.26	1.20	0.137
50–59	0.55	0.22	1.35	0.193
60+	0.24	0.06	0.93	0.039
Having underlying medical conditions				
No = 0	1.00	–	–	
Yes = 1	0.55	0.25	1.19	0.127
Occupation				
Physician (Reference)	1.00	–	–	
Dentist	2.11	0.64	6.97	0.222
Nurse	0.64	0.15	2.63	0.534
Dental hygienist	1.71	0.43	6.79	0.443
Care worker	1.10	0.19	6.27	0.912
non-Health care worker	1.65	0.49	5.61	0.422
Q9. You may experience fever or swelling at the vaccination site after receiving the COVID-19 vaccine.				
(Strongly) disagree/Neither = 0	1.00	–	–	0.049
(Strongly) agree = 1	0.41	0.17	0.99	
Q10. I am concerned about adverse reactions to the COVID-19 vaccine.				
(Strongly) disagree/Neither = 0	1.00	–	–	0.013
(Strongly) agree = 1	2.66	1.23	5.74	
Q11. Prevention of COVID-19 in vaccinated persons.				
(Strongly) disagree/Neither = 0	1.00	–	–	0.514
(Strongly) agree = 1	1.37	0.54	3.49	
Q12. Prevent family members and friends of the inoculated person from contracting COVID-19.				
(Strongly) disagree/Neither = 0	1.00	–	–	0.717
(Strongly) agree = 1	0.83	0.31	2.26	
Q13. Prevent the spread of COVID-19 in the vaccinated person’s area.				
(Strongly) disagree/Neither = 0	1.00	–	–	0.520
(Strongly) agree = 1	0.77	0.35	1.71	

*Logistic regression analysis.

^a^Acceptance (Fourth and fifth doses) = 0, hesitant (Third or less doses) = 1.

## Discussion

This was the first study to compare literacy and confidence regarding COVID-19 and its vaccine and to identify the factors associated with COVID-19 vaccine hesitancy among HCWs, including oral health professionals, in Japan. In addition, the study participants were surveyed under the same COVID-19 vaccination setting and in the same community, which means that the results of this study could be presented after excluding confounding factors such as convenience of access to the vaccine and regional characteristics.

The multiple-comparison test results showed that the DHs’ COVID-19 literacy was significantly lower than that of the physicians or nurses. In addition, they were more concerned about the adverse reactions to the vaccine, than the physicians were and less confident about the preventive effect of the vaccine than were the nurses. Previous studies have reported that younger age was positively associated with low COVID-19 vaccine literacy [24, 25]. Moreover, other studies reported that younger age was among the factors associated with the occurrence of reverse effects [26–28]. Therefore, the DHs’ younger age, compared with the physicians and nurses, might have affected their low COVID-19 literacy and high concern about the reverse effects of vaccinations. Inadequate health literacy contributed to the low rate of COVID-19 vaccination among Turkey patients [29]. A systematic review has reported that vaccine literacy plays an important role in determining the level of vaccine hesitancy across various populations [30]. Therefore, DHs should be further educated to improve their COVID-19 literacy and vaccine hesitancy.

More than 95% of the study participants had received more than third dose of the vaccination; however, this percentage was only 67.4% in the Japanese population during the same period [2]. Priority vaccination policies for medical professionals [31] and easy access to vaccination sites may have influenced their high vaccination rates. The mean numbers of vaccine doses for the dentists and DHs were the lowest among the HCWs, and the number of doses was significantly lower for dentists than for physicians after the multiple-comparison test. This suggests that the hesitancy of oral health professionals to administer the vaccine was higher than that of the other HCWs in this study. Most oral health professionals were under 40 years old, whereas the majority of other HCWs were over 40 years old. Therefore, age may affect the number of vaccine doses [28]. Conversely, a previous study [17] conducted in December 2020 compared vaccine hesitancy among HCWs, including dentists in Greece, and reported that dentists were the most accepting of the vaccine, followed by physicians and pharmacists. Differences in countries and survey periods might have caused the difference in the results between the previous and present studies.

Younger age and greater concern about the adverse effects of the vaccine were positively associated with COVID-19 vaccine hesitancy. COVID-19 vaccine hesitancy was consistently associated with younger age, and high concern about the reverse effects of the vaccine were consistently reported in different countries and at different times of the survey, which might mean that they were universal factors for vaccine hesitancy [12]. Some studies have reported that the younger generations’ lower perceived likelihood to experience the negative severe effects of COVID-19 and to experience more severe adverse effects than older adults might contribute to their vaccine hesitancy for COVID-19 [32]. Therefore, these perceptions could affect the association between age group and vaccine hesitancy in this study.

Another factor that was positively associated with hesitancy was low recognition of the possibility of experiencing reverse effects at the vaccination site. In this study, those who had received the third or lower doses were defined as the hesitant group; therefore, the low number of vaccine doses they received might have affected their low recognition rate. Occupation was not a factor in vaccine hesitancy in multivariate analysis, although there were significant differences in the hesitancy between dentists and physicians in the multi-comparison test. However, further studies are needed to compare hesitancy between oral health professionals and other HCWs in different environments, such as other health professional schools, hospitals, and private clinics.

The results of this study revealed that oral health professionals had lower COVID-19 literacy and higher vaccine hesitancy than other HCWs such as physicians and nurses although age might affect in the results. Therefore, it is suggested that oral health professionals, particularly young ones should receive education to improve them. A previous study found that practical exercises, such as simulation role-playing, interprofessional collaboration, and digital literacy, may improve vaccine education [33]. Therefore, such interprofessional education for HCWs may help improve young oral health professionals’ COVID-19 vaccine literacy and hesitancy.

This study has several limitations. First, this survey was conducted after COVID-19 vaccines became widely available. Therefore, a comparison of the literacy and hesitancy regarding the vaccine between this study and previous studies, which were conducted before the vaccine had not been widespread, should be made with caution. Second, the number of vaccine doses was investigated using a self-administered questionnaire, which may not reflect the actual number of doses. Third, the response rate was 76.5%. A study on the response rates of surveys showed that response rates were higher when the survey topic was interesting to respondents [34]. Therefore, the respondents in this study might have been more interested in the vaccine and less hesitant to vaccinations than non-responders. Finally, this survey was conducted only in school cooperation facilities. Further studies in other health professional schools, hospitals, and clinics are needed to demonstrate the differences in vaccine literacy and hesitancy among HCWs.

## Conclusion

Literacy regarding COVID-19 and its vaccine hesitancy was compared among HCWs, including, oral health professionals, under the same COVID-19 vaccination environment in Japan. The results showed that oral health professionals were less literate about COVID-19 than were physicians and nurses. The number of vaccine doses administered by dentists was significantly lower than were those administered by physicians. Younger age and higher concern about the reverse effects of the vaccine were positively associated with greater hesitation to use the vaccine. It is suggested that young HCWs, especially young oral health professionals, should be further educated about COVID-19 and the vaccine compared to other healthcare workers to promote vaccination in healthcare facilities. Further studies are needed to investigate other facilities, such as other healthcare professional schools, hospitals, and clinics, to reveal the differences among HCWs.

## Supplementary information

SI Table 1

## Data Availability

The datasets generated and/or analysed during the current study are not publicly available, as ethics approval was granted on the basis that only the researchers involved in the study could access the identified data but are available from the corresponding author on reasonable request.
