# Supplementary material for: Comparing COVID-19 literacy and vaccine hesitancy among health care workers, including oral health professionals, in Japan
Source: BDJ Open. 2025 Jan 4;11:1. doi: 10.1038/s41405-024-00282-9 (PMC11700177; doi:10.1038/s41405-024-00282-9)
Supplement: Supplementary file 1 — SI Table 1 [file 41405_2024_282_MOESM1_ESM.pdf]

Supplementary Table. Questionnaire used in this study

Q1 Demographic details. Please check one that applies to each question.

a. Age group (years). □ 20–29, □ 30–39, □ 40–49, □ 50–59, □ more than 60 years

b. Gender. □ Male, □ Female

c. Workplace.

□ Hospital, □ Dental school, □ Dental hygiene school, □ Nursing school, □ Elderly care facility

d. Occupation

□ Physician, □ Dentist, □ Nurse (including public health nurse and midwife), □ Dental hygienist, □ Caregiver, □ Nursing assistant, □ Pharmacist, □ Radiologist, □ Physical therapist, □ Occupational therapist, □ Speech pathologist, □ Dental technician, □ Laboratory technician, □ Optometrist, □ Dental technician, □ Speech-language pathologist, □ Optometrist, □ Clinical psychologist, □ Clinical engineer, □ Administrative staff

□ Academic staff (position not included above), □ Other: ________________________

Q2. Do you have any underlying medical conditions?

□ Yes (Hypertension, diabetes, asthma, heart disease, chronic respiratory disease, chronic kidney disease, liver disease, immune-compromising diseases)

□ No

Q3-a. Have you ever received the novel coronavirus (COVID-19) vaccine?

□ Yes, □ No

Q3-b. If you answered "Yes" to Q3-a, how many times have you received the vaccine? Please check one that applies.

□ 1 time, □ 2 times, □ 3 times, □ 4 times, □ 5 times

Q4. What do you think about the following information regarding novel coronavirus (COVID-19) infection? Please check one that applies to each question.

a. I know a lot about COVID-19.

□ strongly disagree, □ disagree, □ neither agree nor disagree, □ agree, □ strongly agree

b. Anyone with COVID-19 will have symptoms.

□ strongly disagree, □ disagree, □ neither agree nor disagree, □ agree, □ strongly agree

c. Many people with COVID-19 have mild illnesses.

□ strongly disagree, □ disagree, □ neither agree nor disagree, □ agree, □ strongly agree

d. COVID-19 is more severe in people over 65 years old and those with chronic illnesses.

□ strongly disagree, □ disagree, □ neither agree nor disagree, □ agree, □ strongly agree

e. COVID-19 easily spreads from person to person.

□ strongly disagree, □ disagree, □ neither agree nor disagree, □ agree, □ strongly agree

f. I am worried about getting COVID-19.

□ strongly disagree, □ disagree, □ neither agree nor disagree, □ agree, □ strongly agree

g. I may get a new type of COVID-19.

□ strongly disagree, □ disagree, □ neither agree nor disagree, □ agree, □ strongly agree

h. Once you have COVID-19, you cannot get it again.

□ strongly disagree, □ disagree, □ neither agree nor disagree, □ agree, □ strongly agree

Q5. What do you think about the following information regarding novel coronavirus (COVID-19) vaccine? Please check one that applies to each question.

a. Prevention of COVID-19 in vaccinated persons.

□ strongly disagree, □ disagree, □ neither agree nor disagree, □ agree, □ strongly agree

b. Prevent family members and friends of the inoculated person from contracting COVID-19.

□ strongly disagree, □ disagree, □ neither agree nor disagree, □ agree, □ strongly agree

c. Prevent the spread of COVID-19 in the vaccinated person's area.

□ strongly disagree, □ disagree, □ neither agree nor disagree, □ agree, □ strongly agree

d. I am concerned about adverse reactions to the COVID-19 vaccine.

□ strongly disagree, □ disagree, □ neither agree nor disagree, □ agree, □ strongly agree

e. You may experience fever or swelling at the vaccination site after receiving the COVID-19 vaccine.

□ strongly disagree, □ disagree, □ neither agree nor disagree, □ agree, □ strongly agree
